# Integrated Analysis of miRNA and mRNA Expression Profiles Reveals Functional miRNA-Targets in Development Testes of Small Tail Han Sheep

**DOI:** 10.1534/g3.118.200947

**Published:** 2018-12-17

**Authors:** Man Bai, Limin Sun, Chao Jia, Jiarong Li, Yue Han, Hang Liu, Yang Chen, Huaizhi Jiang

**Affiliations:** * College of Animal Science and Technology, Jilin Agricultural University, Changchun 130118, China; † College of Animal Husbandry and Veterinary Medicine, Shenyang Agricultural University, Shenyang 110866, China

**Keywords:** Small Tail Han Sheep, testis development, spermatogenesis, miRNAs, mRNAs

## Abstract

Small Tail Han Sheep is a highly valued local breed in China because of their precocity, perennial estrus, and high fecundity. The average annual lambing rate of ewes is as high as 180–270%, the semen of ram has characteristics of high yield, high density, and good motility. To reveal the key miRNAs and miRNA-targets underlying testis development and spermatogenesis in male Small Tail Han Sheep, integrated analysis of miRNA and mRNA expression profiles in 2-, 6-, and 12-month-old testes was performed by RNA-seq technology and bioinformatics methods. The results showed that total of 153 known sheep miRNAs and 2712 novel miRNAs were obtained in 2-,6 - and 12-month-old Small Tail Han Sheep testes; 5, 1, and 4 differentially expressed (DE) known sheep miRNAs, and 132, 105, and 24 DE novel miRNAs were identified in 2- *vs.* 6-, 6- *vs.* 12-, and 2- *vs.* 12-month-old testes, respectively. We combined miRNA results of this study and the mRNA results obtained in our previous study to predict the target mRNAs of DE known sheep miRNAs; 131, 10, and 15 target mRNAs of DE known sheep miRNAs and 76, 1, and 11 DE miRNA–targets were identified in the three groups, respectively. GO and KEGG analyses showed that: in 2- *vs.* 6-month-olds, the target genes of DE known sheep miRNAs were involved in 100 biological processes and 11 signaling pathways; in 6- *vs.* 12-month-olds, the target genes of DE known sheep miRNAs were involved in 4 biological processes; and in 2- *vs.* 12-month-olds, the target genes of DE known sheep miRNAs were involved in 17 biological processes and 4 signaling pathways. Three miR–target regulatory networks were constructed based on these DE miRNA–targets. The key miRNA-Targets involved in testis development and spermatogenesis were screened. 6 known sheep miRNAs and 6 novel miRNAs were selected to validate the accuracy of miRNA sequencing data by qRT-PCR. The binding sites of oar-miR-379-5p with *WNT8A* was validated by a dual luciferase reporter gene detection system.

In recent years, the sheep industry has developed rapidly. Reproductive efficiency has a major impact on profitability. At present, improving the reproductive efficiency of sheep is an issue of great concern for farmers and herdsmen. Reproductive traits typically have low to medium heritability, the speed of genetic progress obtained by traditional breeding method is slow and the effect is not stable (Abdoli *et al.* 2016). This can be improved by molecular genetic marker and marker-assisted selection (Abdoli *et al.* 2013). Sheep reproductive traits are influenced by many factors, such as genetic background, age, season, environment, and nutrition (Cahill 1981; Makawi *et al.* 2007; Sezenler *et al.* 2011; Pickering *et al.* 2012; Canché *et al.* 2016). Reproductive traits are quantitative traits, which could be controlled by lots of minor genes or by some major genes (Drouilhet *et al.* 2010). Focusing on development of reproductive traits will have a long term effect on profitability in the sheep production (Pramod *et al.* 2013). A lot of researches have demonstrated that high fecundity of ewes is mainly related to some major fecundity genes, such as *BMP15*, *BMPR1B*, *GDF9*, *B4GALNT2*, *ESR1*, *FSHR*, *POU1F1*, *OLKUSKA* and *BELLE-ILE* (Liu *et al.* 2014; Abdoli *et al.* 2016). There is little research to select the molecular markers in ram. Small Tail Han Sheep is a highly valued local breed in China that has excellent breeding characteristics, such as large size, early sexual maturity, perennial estrus, and multiple births (Chu *et al.* 2011). The average annual lambing rate of ewes is as high as 180–270%, and the semen of ram has characteristics of high yield, high sperm density, and good sperm motility. Male Small Tail Han Sheep can be used as a good research subject. Sperm quality is closely related to testis tissue structure, testis development and spermatogenesis are obvious factors that affect male reproductive efficiency (Gong *et al.* 2013; Zhu *et al.* 2016). Therefore, understanding the molecular regulatory mechanisms and finding out the molecular marker underlying testis development and spermatogenesis in Small Tail Han Sheep will provide theoretical basis for improving male sheep reproductive efficiency.

With the wide application of molecular biology technology in animal genetics and breeding, it is possible to systematically clarify the molecular regulatory mechanisms underlying gene functions (Miao and Qin 2015). Gene regulation is at the core of all biological behaviors and phenotypes, which is a multi-mode spatial and temporal dynamic process and mainly regulated at transcriptional and post-transcriptional levels (Wang *et al.* 2016). Generally, transcriptome refers to the sum of all RNAs transcribed by an organism in a particular environment or physiological state, including mRNA and noncoding RNA (such as rRNA, tRNA, microRNA, siRNA, piRNA, circle RNA, and lncRNA). miRNAs are important noncoding RNAs that regulate gene expression and participate in many biological and metabolic processes, such as growth, development, reproduction, and disease, by specifically binding to 3′untranslated regions (UTRs) of mRNA to degrade a target gene or inhibit target gene translation (Ambros 2004; Ran *et al.* 2014). Increasing evidence has shown that miRNAs play key roles in testis development and spermatogenesis (Kotaja 2014; Pratt and Calcatera 2016). Testis development and spermatogenesis have obvious stage specificity, miRNA expression changes with testis development and spermatogenic cell development (Zhang *et al.* 2010; Lian *et al.* 2012; Wu *et al.* 2014; Kasimanickam and Kasimanickam 2015; Kasimanickam and Kasimanickam 2015; Tariq *et al.* 2016). To date, many miRNAs have been identified and studied in different species, such as humans, mice, pig, cattle and chicken (Yan *et al.* 2007; Zhang *et al.* 2010; Huang *et al.* 2011; Luo *et al.* 2015; Wang and Xu 2015; Wu *et al.* 2017b). However, miRNAs identification and molecular mechanism exploration has not been investigated in sheep testis as in other livestock species. Therefore, it is necessary to investigate miRNA and mRNA expression profiles to understand the molecular regulatory mechanism and identify the key miRNA-targets underlying testes development and spermatogenesis in Small Tail Han Sheep.

In our previous publication, histological structure and mRNA expression profiles of 2-, 6-, and 12-month-old Small Tail Han Sheep testes were investigated using hematoxylin–eosin staining and RNA-seq technology, respectively (Bai *et al.* 2017). In this study, we analyzed miRNA expression profiles in 2-, 6-, and 12-month-old Small Tail Han Sheep testes, integrated the miRNA expression profile in this study and the mRNA expression profile obtained in our previous study to construct miRNA–target regulatory networks, selected the key miRNAs and their target mRNAs involved in testis development and spermatogenesis in Small Tail Han Sheep. These results of our study can help to identify the molecular markers affecting reproductive efficiency of male Small Tail Han Sheep and provide theory evidence for further finding out the molecular markers affecting male sheep reproductive ability.

## MATERIALS AND METHODS

### Sample preparation

Two-, 6-, and 12-month-old male Small Tail Han Sheep from the nucleus herd bred by Songyuan Sheng Hua Animal Husbandry Co. Ltd (Jilin, China) were used for this experiment. Three different rams of each age were selected for sample collection. Left testis of each ram was collected by surgical operation and immediately frozen in liquid nitrogen. Intratesticular tissues were used for total RNA extraction. Samples were the same as those used in our previous study (Bai *et al.* 2017).

### miRNA library construction and sequencing

Total RNA was extracted from testis tissues using Trizol Reagent (Invitrogen, USA). RNA integrity number (RIN) and RNA quality were determined using Agilent Tapestation 2200 (Agilent, USA) and NanoDrop 2000 (Thermo Scientific, USA) instruments. RNA samples with RIN ≥6.0 were considered acceptable for miRNA library construction. The miRNA library of each sample was prepared using an Ion Total RNA-seq Kit v2 (Life Technologies, USA) following the manufacturer’s instructions. Briefly, 13-34nt RNAs were captured and enriched using magnetic beads with oligo (dt); subsequently, 13-34nt RNAs were fractionated into short fragments using RNase III and an Ion adaptor. The short RNA fragments were ligated with 3′ and 5′ adaptors. Then, the short mRNA fragments were reverse-transcribed and amplified to synthetize double-stranded cDNA. Emulsion PCR was performed using the miRNA cDNA library as the template. RNA-seq was conducted on an ABI Ion Proton instrument by NovelBio Bio-Pharm Technology Co. Ltd (Shanghai, China). Raw sequencing data were assessed by Fast-QC (http://www.bioinformatics.babraham.ac.uk/projects/fastqc) (Ramayo-Caldas *et al.* 2012).

### DE miRNA analysis

The raw sequencing reads were filtered, and the clean reads were mapped to miRBase 21.0 (http://www.mirbase.org/) to identify known sheep miRNAs (oar-miRNAs). Then, the remaining clean reads that were not mapped to sheep miRBase were mapped to sheep genome Oar3.1 to predict sheep novel miRNAs by miRDeep 2.0.0.8. The remaining clean reads that did not map to sheep genome Oar3.1 were mapped to human, rat, and mouse miRBase using the BWA algorithm to predict novel miRNAs. Only the mapped reads were used for miRNA expression analysis. miRNA expression levels were quantified by the reads per kilobase of transcript per million mapped reads (RPKM) method. The RPKM value was used to calculate expression, and the upper-quartile algorithm was used to correct expression to help produce accurate results for some miRNAs that occurred in low abundance. The DE miRNAs were identified by the EB-Seq algorithm (criteria:|log_2_^FoldChange^|>0.585; false discovery rate, FDR < 0.05); FDR was calculated to correct the P-value (Wright and Simon 2003).

### Venn, Series-cluster and hierarchical clustering analysis of DE miRNAs

Venn analysis was performed to visualize the DE mRNA and miRNA changes by stage using Microsoft Excel 2016. Series-cluster analysis was performed to identify the expression profiles of miRNAs using the STEM program (http://www.cs.cmu.edu/∼jernst/st/). The DE miRNAs in 2- *vs.* 6-, 6- *vs.* 12-, and 2- *vs.* 12-month-old testes were categorized into 8 expression profiles. Hierarchical cluster analysis was performed with Cluster 3.0 and Tree View 1.6 programs (http://rana.lbl.gov/eisen).

### Target prediction of DE miRNAs and integrative analysis

The TargetScan and miRanda algorithms were used to predict putative target mRNAs of DE miRNAs. The putative target mRNAs of DE miRNAs were crossed with DE mRNAs in 2- *vs.* 6-, 6- *vs.* 12-, and 2- *vs.* 12-month-old testes, respectively, which were obtained in our previous study (Bai *et al.* 2017). Negative correlation analysis was performed to determine the relation of DE miRNAs and mRNAs. Pearson correlation coefficients were used to determine whether the expression levels of each particular miRNA and its target mRNAs were negatively correlated (correlation < 0, *P* < 0.05, FDR < 0.05). If a miRNA and its target mRNA were negatively correlated, the mRNA was identified as a candidate target of miRNA.

### Gene Ontology (GO) and Kyoto Encyclopedia of Genes and Genomes (KEGG) pathway analyses

The GO database (http://geneontology.org/) was used to analyze the main functions of candidate target mRNAs. Biological process (BP) terms were annotated for candidate target mRNAs. Similarly, the KEGG database (http://www.genome.jp/kegg/) was used to analyze the pathways in which the candidate target mRNAs are involved. Fisher’s exact and χ^2^ tests were used to classify the significant GO terms and pathways of DE mRNAs (*P* < 0.05), and FDR was calculated to correct the P-value (Wang *et al.* 2014). All of the candidate target mRNAs were analyzed using the GO and KEGG pathway databases.

### miRNA–target regulatory network construction

To identify all possible miRNA–mRNA interactions, miRNA–target regulatory networks were built with Cytoscape 2.8.3. The expression values for DE miRNAs and their candidate target mRNAs were imported into Cytoscape to generate and visualize functional networks.

### RNA-seq data validation

Six known sheep miRNAs and 6 novel mRNAs were randomly selected and analyzed to validate RNA-seq data using qRT-PCR. Synthesized cDNA was used as the template for qRT-PCR. qRT-PCR amplification for miRNAs was performed using the miRcute Plus miRNA qPCR Detection Kit (FP411, TIANGEN BIOTECH, China). The 20-µl reaction system contained 10µl 2×miRcute Plus miRNA Premix, 0.4 µl universal reverse primer (200nM) provided by the kit, 0.4 µl miRNA sequence-specific forward primer (200nM), 2µl cDNA, and 7.2 µl ddH_2_O. The reaction conditions were 95° for 15 min, and 40 cycles of 94° for 20s, 60° for 34s; the reaction was completed with a melting curve analysis according to the manufacturer’s instructions. qRT-PCR was performed on a Bio-Rad CFX96 system (Bio-Rad, USA). All qRT-PCRs were completed with a melting curve analysis. The 2^−ΔΔCt^ method and internal control gene U6 was used to calculate miRNA expression levels. Specific primers were designed and synthesized by Sangon Biotech (Shanghai, China); primer information is shown in Table 1.

**Table 1:** qRT-PCR primer information

Name	Forward primer (5′ to 3′)	Reverse primer (5′ to 3′)
hsa-miR-3529-5p	ACGAGGTAGACTGGGATTTGTTGT T	Universal reverse*
hsa-miR-6820-3p	TGTGACTTCTCCCCTGCCACAG	Universal reverse*
hsa-miR-3615	CTCTCGGCTCCTCGCGGC	Universal reverse*
mmu-miR-1957b	CGCGTGGAATGTAACTCAGTGGTA T	Universal reverse*
mmu-miR-182-3p	CGGTGGTTCTA ACTTGCCAA CT	Universal reverse*
mmu-miR-1929-3p	CAGCTCATGGAGACCTAGGTGG	Universal reverse*
oar-miR-379-5p	CTGGTAGACTATGGAACGTAGGC	Universal reverse*
oar-miR-665-3p	CCAGTAGGCCGAGGCCCC	Universal reverse*
oar-miR-200b	ACGCTAATACTGCCTGGTAATGATG	Universal reverse*
oar-miR-409-5p	CGAATGTTGCTCGGTGAACCCC	Universal reverse*
oar-miR-495-3p	AGCGAAACAAACATGGTGCACTTCTT	Universal reverse*
oar-miR-200c	CGTAATACTGCCGGGTAATGATGG	Universal reverse*
U6	CGAGGATGTGAAGACACCAAGAC	Universal reverse*

* Universal reverse was provided by manufacture (miRcute Plus miRNA qPCR Detection Kit, TIANGEN BIOTECH, China).

### Cell culture

HEK-293T cells were resuscitated and cultured in DMEM containing 10% fetal bovine serum, 1.5 mM L-glutamine, 100U/ml penicillin, and 100μg/ml streptomycin in a 5% CO_2_ saturated humidity incubator at 37°. Adherent cells were passaged daily with 0.05% trypsin–EDTA.

### Plasmid vector construction and transfection

The sequence fragment of *WNT8A*-3′UTR that binds to oar-miR-379-5p (wild-type), *WNT8A*-3′UTR with an oar-miR-379-5p binding site mutation (mutant-type), and positive control (miRNA inhibitor) were designed by Target Scan 7.1 and RNA22 v2.0, and synthesized by total gene synthesis. Sequence information of wild- and mutant-type target gene fragments and the miRNA inhibitor fragment are shown in Table 2. The pmirGLO Dual-Luciferase miRNA Target Expression Vector was used to confirm the putative binding site of *WNT8A*-3′UTR with oar-miR-379-5p. pmirGLO was digested by restriction endonuclease *Sac*I and *Xho*I. pmirGLO and target gene fragments were ligated by T4 DNA ligase. 293T cells were washed and incubated with 4ml serum-free medium for 24h. The miRNA (or miRNA inhibitor) was mixed with 250µl serum-free medium and 1.6µg recombinant vector, and Lipofectamine 2000 transfection reagent (Life Technologies, USA) was mixed with 250µl serum-free medium. Then, the 2 mixtures were combined and incubated for 25min at room temperature. The Lipofectamine 2000–miRNA mixture was added to the cells and incubated at 37° for 5h. Subsequently, 500µL DMEM containing 10% fetal bovine serum was added to the cells and incubated in 5% CO_2_ at 37°. The cells were collected after culturing for 24h and 48h. Then, 7 experimental groups were set up: *WNT8A*-WT+mimics NC, *WNT8A*-WT+oar-miR-379-5p, *WNT8A*-MUT+mimics NC, *WNT8A*-MUT+oar-miR-379-5p, PC+mimics NC, PC+ oar-miR-379-5p, and a blank group. There were 3 replicates of each group.

**Table 2:** Sequence information of wild- and mutant-type target gene fragments and positive control fragments

Gene name	Sequence information (5′-3′)
WNT8A-WT	Sense: CGGAAGTTGGCATCTCAAGAAAAACCATAAGCAGGTTCTTTGCAAGTCTACCCTTATCTCTGTTTTGC
	Antisense: TCGAGCAAAACAGAGATAAGGGTAGACTTGCAAAGAACCTGCTTATGGTTTTTCTTGAGATGCCAACTTCCGAGCT
WNT8A-MUT	Sense: CGGAAGTTGGCATCTCAAGAAAAACCATAAGCAGGTTGTAAGCATCAGATGGCTTATCTCTGTTTTGC
	Antisense: TCGAGCAAAACAGAGATAAGCCATCTGATGCTTACAACCTGCTTATGGTTTTTCTTGAGATGCCAACTTCCGAGCT
PC (oar-miR-379-5p inhibitor)	Sense: CGCCTACGTTCCATAGTCTACCAACCGGTGCCTACGTTCCATAGTCTACCAC
	Antisense: TCGAGTGGTAGACTATGGAACGTAGGCACCGGTTGGTAGACTATGGAACGTAGGCGAGCT

### Dual luciferase reporter detection

Luciferase and *Renilla* activities were measured using a Dual-Luciferase Reporter Assay Kit (Promega Corporation, USA) after transfection for 24h and 48h, respectively.

### Statistical analysis

The data were analyzed using SPSS 18.0, and all results were presented as means ± SD. The significance of differences between groups were assessed by ANOVA. Differences were considered statistically significant when *P* < 0.05.

### Data availability

The sequencing data from this study have been submitted to the NCBI Gene Expression Omnibus (http://www.ncbi.nlm.nih.gov/geo), accession number: GSE107803. All data supporting the results of this article are contained within the article and its Supplemental Materials. Table S1, miRNA Sequence Quality Control. Table S2, read mapping summary of known sheep miRNA. Table S3, the DE novel miRNAs and DE sheep known miRNAs in 2- *vs.* 6-, 6- *vs.* 12-, and 2- *vs.* 12-month-old testes. Table S4, the union of DE miRNA in 2- *vs.* 6-, 6- *vs.* 12-, and 2- *vs.* 12-month-old testes. Table S5, predicted target mRNAs of DE known sheep miRNAs. Table S6, DE miRNA-Targets. Table S7, GO-analysis of candidate target mRNAs in 2- *vs.* 6-, 6- *vs.* 12-, and 2- *vs.* 12-month-old testes. Table S8, pathway analysis of candidate target mRNAs in 2- *vs.* 6-, 6- *vs.* 12-, and 2- *vs.* 12-month-old testes. Table S9, GO and pathway terms that WNT8A involved in related to testis development and spermatogenesis. Supplemental figure 1, Hierarchical clustering of DE mRNAs and DE miRNAs in testis samples. Supplemental material available at Figshare: https://doi.org/10.6084/m9.figshare.7473047.

## RESULTS

### RNA-seq data summary

The miRNA sequence quality control results are shown in Supplementary Table S1. A total of 11.3, 9.6, 16.4, 9.2, 5.7, 6.7, 8.7, 9.3, and 23.8 million high-quality clean reads of miRNAs were obtained from 2- (A1, A2, A3), 6- (B1, B2, B3), and 12-month-old (C1, C2, C3) testes, respectively. For each sample, 1.8–11.5% of miRNA reads were mapped to sheep miRbase (Table S2). Because of the limited information contained in sheep miRBase, the miRNA mapping rate was low.

### DE miRNAs and hierarchical clustering analysis

In our previous publication, we obtained 630, 322, and 102 DE mRNAs in 2- *vs.* 6-, 6- *vs.* 12-, and 2- *vs.* 12-month-old testes, respectively; in total, there were 955 DE mRNAs (Bai *et al.* 2017). In this study, we obtained 5, 1, and 4 DE known sheep miRNAs, and 132,105, and 24 DE novel miRNAs in 2- *vs.* 6-, 6- *vs.* 12-, and 2- *vs.* 12-month-old testes, respectively (Table S3). In total, 211 DE miRNAs were identified in 2- *vs.* 6-, 6- *vs.* 12-, and 2- *vs.* 12-month-old testes, as shown in Supplementary Table S4.

**Figure 1:**
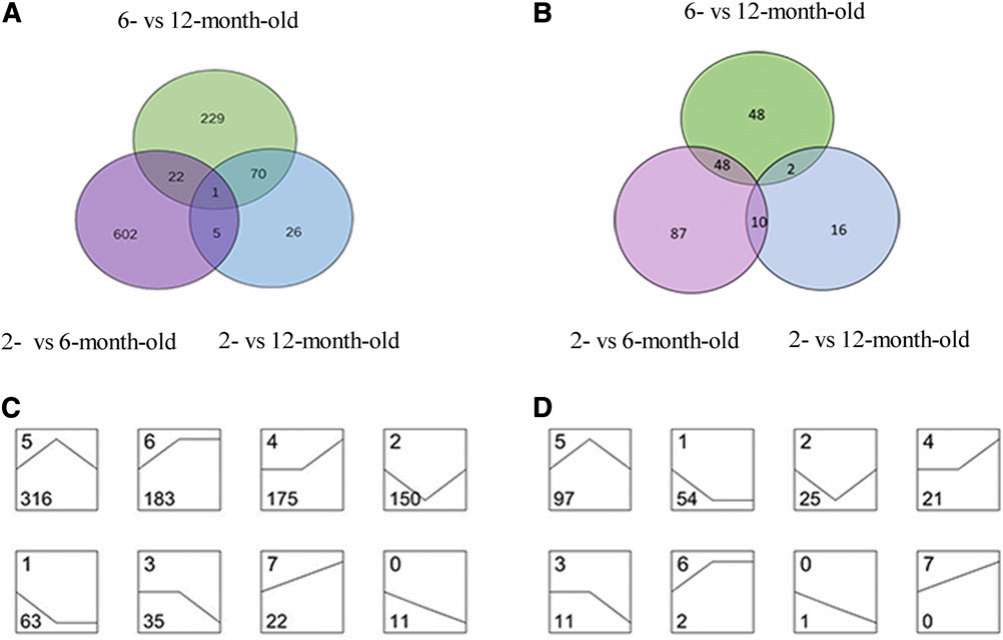
Venn and series-cluster analyses of DE mRNAs and DE miRNAs in 2-, 6-, and 12-month-old testes. a. Venn diagram of DE mRNAs in 2- *vs.* 6-, 6- *vs.* 12-, and 2- *vs.* 12-month-old testes. b. Venn diagram of DE miRNAs in 2- *vs.* 6-, 6- *vs.* 12-, and 2- *vs.* 12-month-old testes. c. Eight profiles of DE mRNAs in 2-, 6-, and 12-month-old testes. d. Eight profiles of DE miRNAs in 2-, 6-, and 12-month-old testes. Profile number is shown at the top left corner of each square. Number of DE mRNAs or miRNAs grouped in each profile is shown at the bottom left corner of each square. Profiles are ordered based on the number of mRNAs or miRNAs assigned.

In Venn analysis, DE mRNA and miRNA changes can be visualized by stage (Figure 1 a b). In series-cluster analysis, we categorized the 955 DE mRNAs and 211 DE miRNAs into 8 possible expression profiles to observe the overall expression tendencies (Figure 1c d). Subsequently, based on these DE mRNAs and DE miRNAs, heat maps were generated by hierarchical clustering analysis (Supplemental figure 1 a b).

### Target prediction of DE miRNAs and integrative analysis

In total, 131, 10, and 15 predicted target mRNAs of DE known sheep miRNAs were obtained from 2- *vs.* 6-, 6- *vs.* 12-, and 2- *vs.* 12-month-old testes, as shown in Table S5. Combined with DE mRNAs, the predicted target mRNAs and DE mRNAs were crossed, and significantly DE target mRNAs were retained. In total, 76, 1, and 11 candidate target mRNAs of DE known sheep miRNAs were identified in 2- *vs.* 6-, 6- *vs.* 12-, and 2- *vs.* 12-month-old testes, respectively (Table S6). For some DE miRNAs, only one target mRNA was identified, however, most of DE miRNAs can target several mRNAs. In addition, several miRNAs can target one mRNA. For example, oar-miR-379-5p targeted 16 different mRNAs; however, GRIK5 is targeted by 2 miRNAs (oar-miR-409-3p and oar-miR-665-3p).

### Candidate target mRNA GO analysis

**Figure 2:**
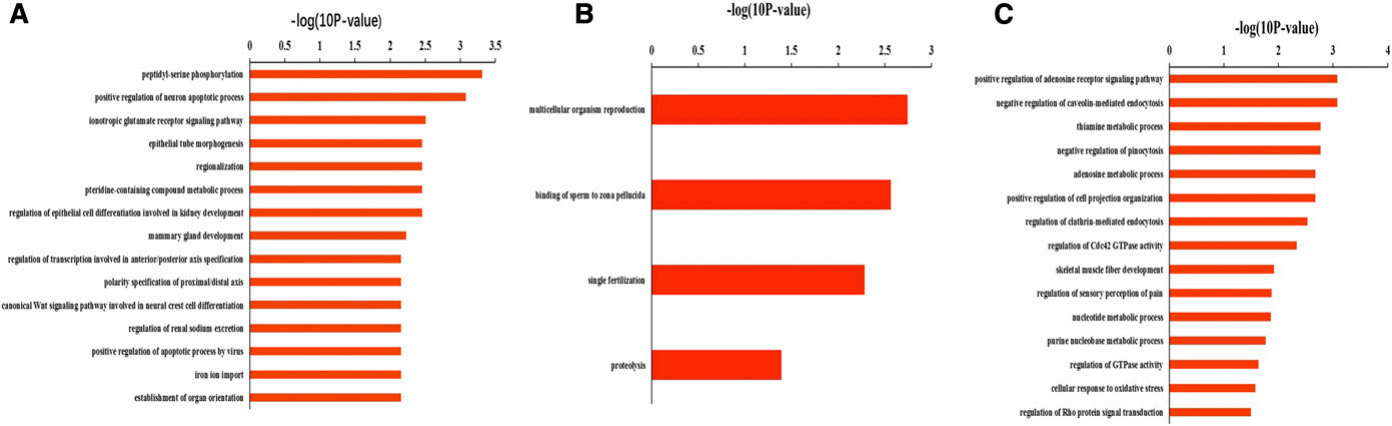
Top 15 BP terms of candidate target mRNAs. a. Two- *vs.* 6-month-old testes; b. 6- *vs.* 12-month-old testes; c. 2- *vs.* 12-month-old testes; *P* < 0.05.

The GO analysis results are shown in Table S7. In 2- *vs.* 6-month-old testes, the candidate target mRNAs were significantly enriched in 100 BP terms, such as apoptotic process, meiosis I, regulation of acrosome reaction, protein phosphorylation, and epithelial cell maturation; the top 15 BP terms are shown in Figure 2 a: peptidyl-serine phosphorylation, positive regulation of neuron apoptotic process, ionotropic glutamate receptor signaling pathway, epithelial tube morphogenesis, regionalization, pteridine-containing compound metabolic process, regulation of epithelial cell differentiation involved in kidney development, mammary gland development, regulation of transcription involved in anterior/posterior axis specification, polarity specification of proximal/distal axis, canonical WNT signaling pathway involved in neural crest cell differentiation, regulation of renal sodium excretion, positive regulation of apoptotic process by virus, iron ion import, and establishment of organ orientation. In 6- *vs.* 12-month-old testes, the candidate target mRNAs were significantly enriched in 4 BP terms, as shown in Figure 2 b: multicellular organism reproduction, binding of sperm to zona pellucida, single fertilization, and proteolysis. In 2- *vs.* 12-month-old sheep testes, the candidate target mRNAs were significantly enriched in 17 BP terms, such as adenosine metabolic process, nucleotide metabolic process, purine nucleobase metabolic process, cellular response to oxidative stress, and RNA processing; the top 15 BP terms are shown in Figure 2 c: positive regulation of adenosine receptor signaling pathway, negative regulation of caveolin-mediated endocytosis, thiamine metabolic process, negative regulation of pinocytosis, adenosine metabolic process, positive regulation of cell projection organization, regulation of clathrin-mediated endocytosis, regulation of Cdc42 GTPase activity, skeletal muscle fiber development, regulation of sensory perception of pain, nucleotide metabolic process, purine nucleobase metabolic process, regulation of GTPase activity, cellular response to oxidative stress, and regulation of Rho protein signal transduction.

### Candidate target mRNA pathway analysis

**Figure 3:**
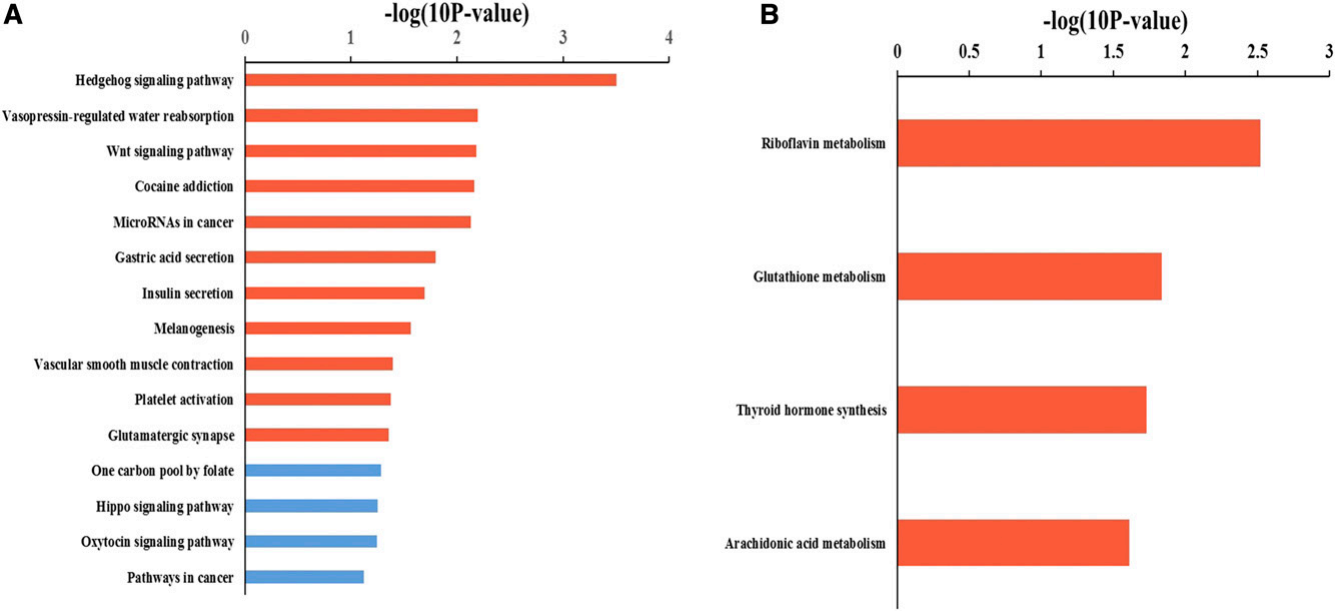
Top 15 pathways of candidate target mRNAs. a. Two- *vs.* 6-month-old testes; b. 2- *vs.* 12-month-old testes; red terms represent *P* < 0.05.

**Figure 4:**
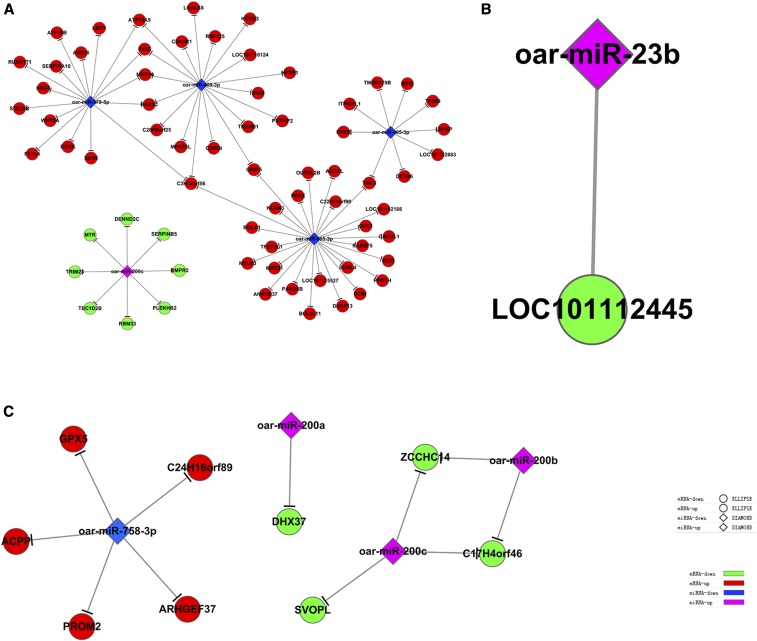
miRNA-Target regulatory networks in 2- *vs.* 6-, 6- *vs.* 12-, and 2- *vs.* 12-month-old testes. a. Two- *vs.* 6-month-old testes; b. 6- *vs.* 12-month-old testes; c. 2- *vs.* 12-month-old testes.

The results of pathway analysis are shown in Table S8 and Figure 4. In 2- *vs.* 6-month-old testes, the candidate target mRNAs were significantly involved in 11 pathways, which are shown in Figure 3 a: Hedgehog signaling pathway, vasopressin-regulated water reabsorption, WNT signaling pathway, cocaine addiction, microRNAs in cancer, gastric acid secretion, insulin secretion, melanogenesis, smooth muscle contraction, platelet activation, and glutamatergic synapse. In 6- *vs.* 12-month-old testes, there was only one candidate target mRNA of DE known sheep miRNAs that was not enriched in any signaling pathway. In 2- *vs.* 12-month-old testes, the candidate target mRNAs were significantly involved in 4 pathways, as shown in Figure 3 b: riboflavin metabolism, glutathione metabolism, thyroid hormone synthesis, and arachidonic acid metabolism.

### miRNA–target regulatory networks

miRNA–target regulatory networks in 2- *vs.* 6-, 6- *vs.* 12-, and 2- *vs.* 12-month-old testes were generated as shown in Figure 4. miRNA–target regulatory networks contained networks of downregulated miRNAs with upregulated mRNAs and networks of upregulated miRNAs with downregulated mRNAs in different testis development stages. In 2- *vs.* 6-month-old testes, 4 downregulated miRNAs with 60 upregulated mRNAs and 1 upregulated miRNA with 8 downregulated mRNAs were identified (Figure 4 a); in 6- *vs.* 12-month-old testes, 1 upregulated miRNA with 1 downregulated mRNA was identified (Figure 4 b); and in 2- *vs.* 12-month-old testes, 3 upregulated miRNAs with 3 downregulated mRNAs and 1 downregulated miRNA with 5 upregulated mRNAs were identified (Figure 4 c).

### RNA-seq data validation

**Figure 5:**
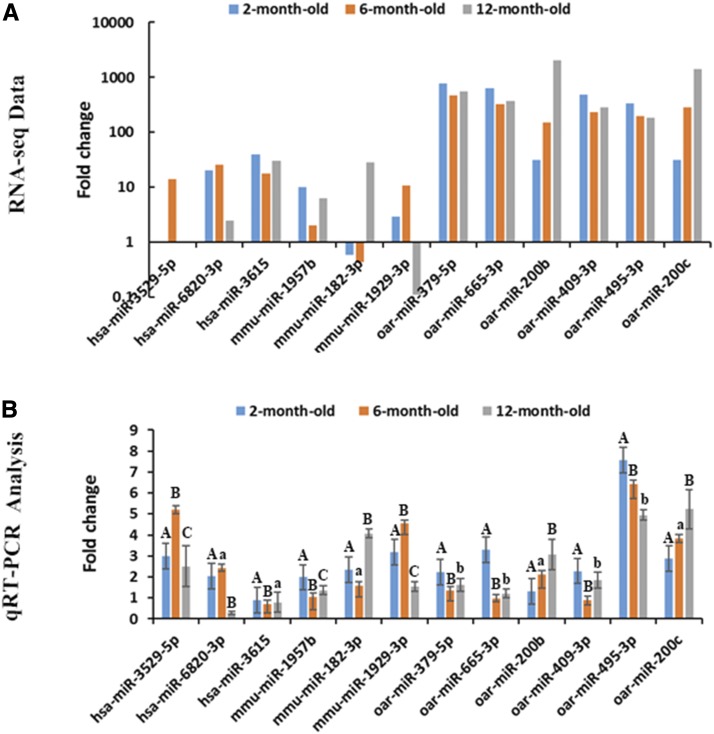
qRT-PCR validation of RNA-seq data for miRNAs. a. RNA-seq data. Values were calculated and normalized by EB-Seq algorithm if the fold changes were >1.5 and FDR < 0.05. b. RT-PCR analysis of 6 novel miRNAs and 6 known miRNAs. The fold change in each time point was relative to 2 months. The expression values were calculated by the 2^−ΔΔCt^ method. Different capital letters represent extremely significant differences (*P* < 0.01); different letters indicate significant differences (*P* < 0.05), whereas the same letters indicate no significant differences (*P* > 0.05).

Six known sheep miRNAs (oar-miR-379-5p, oar-miR-665-3p, oar-miR-409-5p, oar-miR-495-3p, oar-miR-200b, and oar-miR-200c) and six novel miRNAs (hsa-miR-3529-5p, hsa-miR-6820-3p, hsa-miR-3615, mmu-miR-1957b, mmu-miR-182-3p, and mmu-miR-1929-3p) were randomly selected to validate RNA-seq data. qRT-PCR was performed to detect DE miRNA expression. The qRT-PCR results consistent with RNA-seq data (Figure 5 a b).

### oar-miR-379-5p targets WNT8A

**Figure 6:**
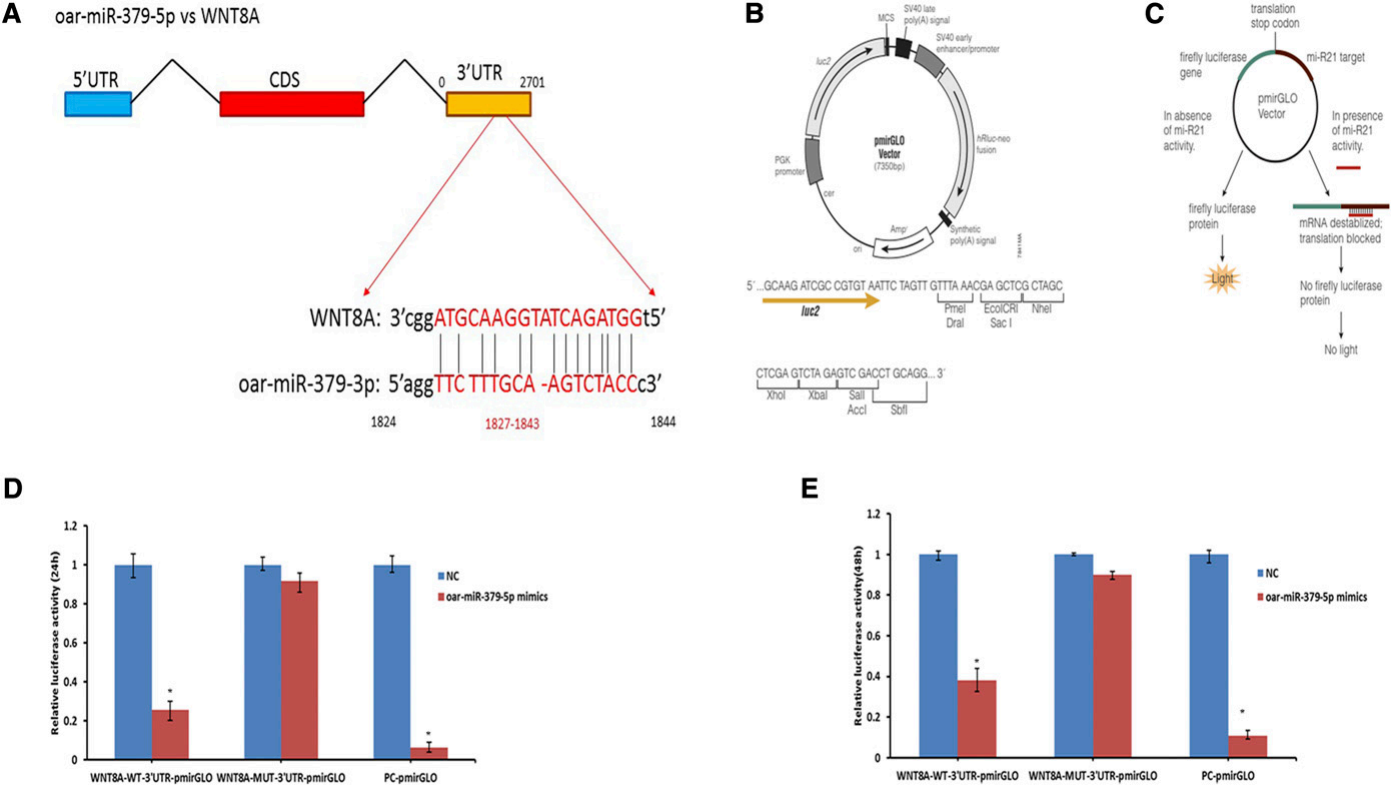
Detection of interactions between oar-miR-379-5p and *WNT8A* by dual luciferase reporter system. a. Binding site sequence of miRNA and target gene *WNT8A*. b. Structure of pmirGLO plasmid vector. c. Working principle of pmirGLO plasmid vector. The dual luciferase reporter gene was inserted the 3′UTR sequence of the target gene, which can bind with miRNA into the downstream of the luciferase gene in pmirGLO plasmid vector, the translation of luciferase from firefly will be inhibited and the luciferase activity will eventually decrease when endogenous miRNA or introduced exogenous miRNA are combined with the inserted 3′UTR sequence. d. Relative luciferase activity after transfecting oar-mir-3799-5p and *WNT8A* recombinant plasmid for 24h. e. Relative luciferase activity after transfecting oar-mir-3799-5p and *WNT8A* recombinant plasmid for 48h.

According to GO and pathway analysis, 78 target mRNAs of 10 DE known sheep miRNAs in 3 groups were further studied to identify the testis development- and spermatogenesis-related biological processes and signaling pathways in which these target mRNAs were involved. It was found that *WNT8A* was closely related to tissue development processes, such as multicellular organismal development, cell morphogenesis, and cell fate commitment, and signaling pathways such as WNT, melanogenesis, and Hedgehog signaling pathways; the GO and pathway terms associated with *WNT8A* are shown in Table S9. Thus, oar-miR-379-5p and *WNT8A* were selected to verify their targeted regulatory relationship. The binding site of *WNT8A*-3′UTR with oar-miR-379-5p is shown in Figure 6 a. pmirGLO plasmid vector and its working principle are shown in Figure 6 b c. Luciferase assays were performed to confirm the potential binding between oar-miR-379-5p and *WNT8A* to further validate the interaction of miRNA–mRNA. The luciferase activity of the *WNT8A* receptor decreased 74.2% and 61.8% upon co-transfection with oar-miR-379-5p mimics (*P* < 0.05) compared with co-transfection with negative control mimics at 24h and 48h, respectively (Figure 6 d e), which indicated that oar-miR-379-5p can directly target *WNT8A*-3′UTR.

## DISCUSSION

Sheep reproductive efficiency is important for livestock husbandry; testis development and spermatogenesis are key factors influencing male sheep reproductive efficiency, many functional genes regulate testis development and spermatogenesis (Gong *et al.* 2013; Ding *et al.* 2016). The aim of this study was to identify the key miRNAs and functional miRNA-targets in developing testis of Small Tail Han Sheep using integrated analysis of miRNA and mRNA expression profiles. The analysis will help us understanding the molecular regulating mechanism in testis development and reveal the molecular markers related to reproductive efficiency of male Small Tail Han Sheep.

In this study, 2-, 6-, and 12-month-old corresponding to infant, adolescent and adult stages in male Small Tail Han Sheep presenting the dynamic process of testis development. Only 5, 1, and 4 DE known sheep miRNAs were identified in 2- *vs.* 6-, 6-vs 12-, and 2-vs 12-month-old testes, respectively, which is because of the limited information in sheep miRBase. Novel miRNAs were predicted from sheep genome, human, rat, and mouse miRBase; therefore, more DE novel miRNAs were identified than DE known sheep miRNAs. DE novel miRNAs can help to enrich our knowledge about the key miRNAs involved in sheep testis development and provide new references for the sheep miRBase. The DE known sheep miRNAs were reported in previous studies, miR-409-3p, miR-665-3p, miR-379-5p, miR-495-3p, miR-23b, and miR-758-3p are all involved in cell proliferation, differentiation, migration, and apoptosis (Weng *et al.* 2012; Dong *et al.* 2016; Chen *et al.* 2017a; Chen *et al.* 2017b; Jiang *et al.* 2017; Xie *et al.* 2017). Based on our results, we can infer that oar-miR-409-3p, oar-miR-665-3p, oar-miR-379-5p, oar-miR-495-3p, oar-miR-23b, and oar-miR-758-3p may play key roles in growth, proliferation, and apoptosis of testis cells. oar-miR-200a, oar-miR-200b, and oar-miR-200c belong to the miR-200 family, which plays a key role in epithelial–mesenchymal transition (Mongroo and Rustgi 2010; Pan *et al.* 2017). It can be conferred that oar-miR-200a, miR-200b, and miR-200c may be associated with seminiferous epithelium development. Currently, these miRNAs are mainly investigated in the context of invasion and migration of cancer, the specific functions of these miRNAs in sheep testes need to be further explored and validated.

GO and KEGG pathway analyses can help elucidate the functions and pathways that candidate target genes involved in. As our results shown: in 2- *vs.* 6-month-old testes, most of the GO terms were related to spermatogenesis (such as apoptotic process, meiosis I, and acrosome reaction), and most of the pathways were related to tissue development (such as Hedgehog and WNT signaling pathways); in 6- *vs.* 12-month-old testes, GO terms were related to male reproductive processes (multicellular organism reproduction, binding of sperm to zona pellucida, single fertilization, and proteolysis). In 2- *vs.* 12-month-old sheep testes, most GO terms and pathways were related to testis metabolic and biosynthesis processes.

Integrative analysis of miRNA and mRNA expression profiles can help elucidate molecular regulatory mechanisms (Miao and Qin 2015). This study is the first attempt to identify key miRNA–targets by integrated analysis of miRNA and mRNA expression profiles in Small Tail Han Sheep testes. Regulatory networks of DE known sheep miRNAs and their target mRNAs can help us more precisely identify the functions of these miRNAs in testis development and spermatogenesis in sheep. In this study, 3 miRNA–target networks were constructed and several miRNA–targets were identified. According to GO and pathway analysis, *WNT8A* (the target of oar-miR-379-5p) was determined to be involved in biological processes such as multicellular organismal development, endoderm development, and palate development, and signaling pathways such as the WNT, Hedgehog, and Hippo signaling pathways. It was previously reported that miR-379 is a key miRNA that inhibits proliferation, invasion, and migration of tumor cells, and plays an important role in tumor occurrence and development; miR-379 can also inhibit proliferation, invasion, and migration of vascular smooth muscle cells by targeting *IGF-1* and regulate breast and liver cancer processes by inhibiting the expression of *Cyclin B1* and *IGF-1R* (Khan *et al.* 2013; Li *et al.* 2017a). Studies have shown that miR-379-5p expression was significantly downregulated in cancer tissues, such as liver, bladder, and breast cancer tissues, and closely related to tumor stage; moreover, miR-379-5p can also inhibit the transformation and metastasis of endothelium to mesenchymal cells in liver cancer (Chen *et al.* 2016; Wu *et al.* 2017a). To date, miR-379 has been widely studied in human cancer, and its research on reproductive organ development has not been reported yet. *WNT8A* is a member of the WNT ligand family, which participates in the WNT, Hedgehog, and Hippo signaling pathway. Most studies reported that *WNT8A* gene plays a vital role on embryonic development and early organ formation (Narayanan 2012; Vendrell *et al.* 2013; Wylie *et al.* 2014; Hino *et al.* 2018). It is reported that the WNT pathway is involved in the major events during testis development, including primordial germ cell specification, proliferation and migration, testis determination, spermatogenesis, and somatic cell regulation (Dong *et al.* 2015). It was also reported that the WNT signaling pathway is involved in male reproductive organ development (Sun and Wang 2003), the Hedgehog signaling pathway is involved in testicular cord and spermatogonial stem cell development (Sahin *et al.* 2014; Li *et al.* 2017b), the Hippo signaling pathway is involved in regulating growth, proliferation, and apoptosis of cells, and regulating proliferation and maintenance of stem cells to control organ size (Pan 2007; Zhang *et al.* 2008). Therefore, it can be inferred that oar-miR-379-5p may be involved in Small Tail Han Sheep testis development and spermatogenesis by downregulated *WNT8A*, which involved in these pathways.

Currently, the dual luciferase reporter gene method is the most convenient method for verifying the relationship between miRNAs and their target mRNAs. The results demonstrated that oar-miR-379-5p can decrease *WNT8A* expression by targeting *WNT8A*-3′UTR. It is the first time to verify that *WNT8A* is the target gene of oar-miR-379-5p. However, the hypothesis that oar-miR-379-5p is involved in testicular development and spermatogenesis by targeting *WNT8A* needs to be further studied and verified. The results of this study will provide theory evidence for identifying the molecular markers in Small Tail Han Sheep testis development and further finding out the molecular markers affecting male sheep reproductive ability.

## CONCLUSION

This study is the first integrative analysis of miRNA and mRNA expression profiles in Small Tail Han Sheep testis development. We identified the key miRNAs and miRNA–targets in 2- *vs.* 6-, 6-vs 12-, and 2-vs 12-month-old testes, which will provide theoretical basis for elucidating the molecular regulatory mechanisms underlying sheep testis development and identifying the biomarkers that affect reproductive efficiency of male Small Tail Han Sheep. Our study is the first to reveal that *WNT8A* was targeted by oar-miR-379-5p. We present a hypothesis that oar-miR-379-5p is involved in sheep testis development and spermatogenesis by targeting *WNT8A* and we will further investigate and verify this hypothesis.

## References

[bib1] Abdoli R. Zamani P. Deljou A. Rezvan H., 2013 Association of BMPR-1B and GDF9 genes polymorphisms and secondary protein structure changes with reproduction traits in Mehraban ewes. Gene 524: 296–303. 10.1016/j.gene.2013.03.133 23583795

[bib2] Abdoli R. Zamani P. Mirhoseini S. Z. Ghavi H. N. Nadri S., 2016 A review on prolificacy genes in sheep. Reprod. Domest. Anim. 51: 631–637. 10.1111/rda.12733 27491513

[bib3] Ambros V., 2004 The functions of animal microRNAs. Nature 431: 350–355. 10.1038/nature02871 15372042

[bib4] Bai M. Sun L. Zhao J. Xiang L. Cheng X., 2017 Histological analysis and identification of spermatogenesis-related genes in 2-, 6-, and 12-month-old sheep testes. Naturwissenschaften 104: 84 10.1007/s00114-017-1505-1 28948304

[bib5] Cahill L. P., 1981 Folliculogenesis in the sheep as influenced by breed, season and oestrous cycle. J. Reprod. Fertil. Suppl. 30: 135–142. 6820051

[bib6] Canché J. E. T. Monforte J. G. M. Correa J. C. S., 2016 Environmental effects on productive and reproductive performance of Pelibuey ewes in Southeastern México. J. Appl. Anim. Res. 44: 508–512. 10.1080/09712119.2015.1102730

[bib7] Chen J. S. Li H. S. Huang J. Q. Dong S. H. Huang Z. J., 2016 MicroRNA-379–5p inhibits tumor invasion and metastasis by targeting FAK/AKT signaling in hepatocellular carcinoma. Cancer Lett. 375: 73–83. 10.1016/j.canlet.2016.02.043 26944318

[bib8] Chen M. Shi J. Zhang W. Huang L. Lin X., 2017a MiR-23b controls TGF-β1 induced airway smooth muscle cell proliferation via direct targeting of Smad3. Pulm. Pharmacol. Ther. 42: 33–42. 10.1016/j.pupt.2017.01.001 28062322

[bib9] Chen Y. Luo D. Tian W. Li Z. Zhang X., 2017b Demethylation of miR-495 inhibits cell proliferation, migration and promotes apoptosis by targeting STAT-3 in breast cancer. Oncol. Rep. 37: 3581–3589. 10.3892/or.2017.5621 28498478

[bib10] Chu M. Jia L. Zhang Y. Jin M. Chen H., 2011 Polymorphisms of coding region of BMPR - IB gene and their relationship with litter size in sheep. Mol. Biol. Rep. 38: 4071–4076. 10.1007/s11033-010-0526-z 21110108

[bib11] Ding H. Luo Y. Liu M. Huang J. Xu D., 2016 Histological and transcriptome analyses of testes from Duroc and Meishan boars. Sci. Rep. 6: 20758 10.1038/srep20758 26865000 PMC4749976

[bib12] Dong C. Du Q. Wang Z. Wang Y. Wu S., 2016 MicroRNA-665 suppressed the invasion and metastasis of osteosarcoma by directly inhibiting RAB23. Am. J. Transl. Res. 8: 4975. 27904698 PMC5126340

[bib13] Dong W. L. Tan F. Q. Yang W. X., 2015 Wnt signaling in testis development: Unnecessary or essential? Gene 565: 155–165. 10.1016/j.gene.2015.04.066 25921962

[bib14] Drouilhet L. Lecerf F. Bodin L. Fabre S. Mulsant P., 2010 Fine mapping of the FecL locus influencing prolificacy in Lacaune sheep. Anim. Genet. 40: 804–812. 10.1111/j.1365-2052.2009.01919.x 19466934

[bib15] Gong W. Pan L. Lin Q. Zhou Y. Xin C., 2013 Transcriptome profiling of the developing postnatal mouse testis using next-generation sequencing. Science China 56: 1–12. 10.1007/s11427-012-4411-y 23269550

[bib16] Hino H. Nakanishi A. Seki R. Aoki T. Yamaha E., 2018 Roles of maternal wnt8a transcripts in axis formation in zebrafish. Dev. Biol. 434: 96–107. 10.1016/j.ydbio.2017.11.016 29208373

[bib17] Huang J. Ju Z. Li Q. Hou Q. Wang C., 2011 Solexa sequencing of novel and differentially expressed microRNAs in testicular and ovarian tissues in Holstein cattle. Int. J. Biol. Sci. 7: 1016–1026. 10.7150/ijbs.7.1016 21912509 PMC3164151

[bib18] Jiang D. Cho W. Li Z. Xu X. Qu Y., 2017 MiR-758–3p suppresses proliferation, migration and invasion of hepatocellular carcinoma cells via targeting MDM2 and mTOR. Biomedicine & pharmacotherapy = Biomedecine & pharmacotherapie 96: 535. 10.1016/j.biopha.2017.10.004 29032337

[bib19] Kasimanickam V. R. Kasimanickam R. K., 2015 Differential expression of microRNAs in sexually immature and mature canine testes. Theriogenology 83: 394–398.e1. 10.1016/j.theriogenology.2014.10.003 25459426

[bib20] Khan S. Brougham C. L. Ryan J. Sahrudin A. O’Neill G., 2013 miR-379 regulates Cyclin B1 expression and is decreased in breast cancer. PLoS One 8: e68753 10.1371/journal.pone.0068753 23874748 PMC3707961

[bib21] Kotaja N., 2014 MicroRNAs and spermatogenesis. Fertil. Steril. 101: 1552–1562. 10.1016/j.fertnstert.2014.04.025 24882619

[bib22] Li K. Wang Y. Zhang A. Liu B. Jia L., 2017a miR-379 Inhibits Cell Proliferation, Invasion, and Migration of Vascular Smooth Muscle Cells by Targeting Insulin-Like Factor-1. Yonsei Med. J. 58: 234–240. 10.3349/ymj.2017.58.1.234 27873518 PMC5122642

[bib23] Li, S., M. Wang, Y. Chen, W. Wang, J. Wu *et al.*, 2017b Role of the Hedgehog Signaling Pathway in Regulating the Behavior of Germline Stem Cells. Stem Cells International, 2017, (2017–8-13) 2017: 1–9. 10.1155/2017/5714608 PMC5572616 28883837

[bib24] Lian C. Sun B. Niu S. Yang R. Liu B., 2012 A comparative profile of the microRNA transcriptome in immature and mature porcine testes using Solexa deep sequencing. FEBS J. 279: 964–975. 10.1111/j.1742-4658.2012.08480.x 22240065

[bib25] Liu Q. Pan Z. Wang X. Wenping H. U. Ran D. I., 2014 Progress on major genes for high fecundity in ewes. Frontiers of Agricultural Science & Engineering 1: 282 10.15302/J-FASE-2014042

[bib26] Luo Z. Liu Y. Chen L. Ellis M. Li M., 2015 microRNA profiling in three main stages during porcine spermatogenesis. J. Assist. Reprod. Genet. 32: 451–460. 10.1007/s10815-014-0406-x 25563581 PMC4363228

[bib27] Makawi S. A. Elsharif B. A. Babiker E. A., 2007 Effect of Season on Freezability of Semen from Two Breed-Types of Desert Sheep in the Sudan. 6: 846–849.

[bib28] Miao X. Qin Q. L. X., 2015 Genome-wide transcriptome analysis of mRNAs and microRNAs in Dorset and Small Tail Han sheep to explore the regulation of fecundity. Mol. Cell. Endocrinol. 402: 32–42. 10.1016/j.mce.2014.12.023 25573241

[bib29] Mongroo P. S. Rustgi A. K., 2010 The role of the miR-200 family in epithelial-mesenchymal transition. Cancer Biol. Ther. 10: 219–222. 10.4161/cbt.10.3.12548 20592490 PMC3040834

[bib30] Narayanan, A., 2012 Molecular Mechanisms of Wnt8a Regulation: Insights Into Vertebrate Mesoderm Development and Patterning. Doctoral Dissertation, Texas A&M University.

[bib31] Pan D., 2007 Hippo signaling in organ size control. Genes Dev. 21: 886–897. 10.1101/gad.1536007 17437995

[bib32] Pan Q. Meng L. Ye J. Wei X. Shang Y., 2017 Transcriptional repression of miR-200 family members by Nanog in colon cancer cells induces epithelial-mesenchymal transition (EMT). Cancer Lett. 392: 26–38. 10.1016/j.canlet.2017.01.039 28163188

[bib33] Pickering N. K. Dodds K. G. Blair H. T. Hickson R. E. Johnson P. L., 2012 Genetic parameters for production traits in New Zealand dual-purpose sheep, with an emphasis on dagginess. J. Anim. Sci. 90: 1411–1420. 10.2527/jas.2011-4163 22100586

[bib34] Pramod R. K. Sharma S. K. Kumar R. Rajan A., 2013 Genetics of ovulation rate in farm animals. Vet. World 6: 833–838. 10.14202/vetworld.2013.833-838

[bib35] Pratt S. L. Calcatera S. M., 2016 Expression of microRNA in male reproductive tissues and their role in male fertility. Reprod. Fertil. Dev. 29: 24 10.1071/RD16293 28278790

[bib36] Ramayo-Caldas Y. Mach N. Esteve-Codina A. Corominas J. Castelló A., 2012 Liver transcriptome profile in pigs with extreme phenotypes of intramuscular fatty acid composition. BMC Genomics 13: 547 10.1186/1471-2164-13-547 23051667 PMC3478172

[bib37] Ran M. Chen B. Yin J. Yang A. Jiang M., 2014 Advances in miRNA research related to testis development and spermatogenesis. Yi Chuan 36: 646–654. 10.3724/SP.J.1005.2014.0646 25076028

[bib38] Sahin Z. Szczepny A. Mclaughlin E. A. Meistrich M. L. Zhou W., 2014 Dynamic Hedgehog signalling pathway activity in germline stem cells. Andrology 2: 267–274. 10.1111/j.2047-2927.2014.00187.x 24574096 PMC4119796

[bib39] Sezenler T. Yildirir M. Ceyhan A. Yüksel M. A. Önal A. R., 2011 The Effects of Body Condition Score and Age of Ewes on the Reproductive Performance in Kivircik, Sakiz and Gokceada Sheep. J. Anim. Sci. Adv. 1: 94–99

[bib40] Sun X. Wang Y., 2003 Wnt signaling pathways in mammalian reproduction. Progress in Biochemistry & Biophysics 30: 180–184.

[bib41] Tariq K. Peng W. Saccone G. Zhang H., 2016 Identification, characterization and target gene analysis of testicular microRNAs in the oriental fruit fly Bactrocera dorsalis. Insect Mol. Biol. 25: 32–43. 10.1111/imb.12196 26486729

[bib42] Vendrell V. Vázquezecheverría C. Lópezhernández I. Alonso B. D. Martinez S., 2013 Roles of Wnt8a during formation and patterning of the mouse inner ear. Mech. Dev. 130: 160–168. 10.1016/j.mod.2012.09.009 23041177

[bib43] Wang J. Lv Q. Li X. Liu Y. Liu K., 2016 Post-transcriptional and translational regulation modulates gene co-expression behavior in more synchronized pace to carry out molecular function in the cell. Gene 587: 163–168. 10.1016/j.gene.2016.04.055 27150569

[bib44] Wang L. Xu C., 2015 Role of microRNAs in mammalian spermatogenesis and testicular germ cell tumors. Reproduction 149: R127–R137. 10.1530/REP-14-0239 25352684

[bib45] Wang, W., M. Meng, Y. Zhang, C. Wei, Y. Xie *et al.*, 2014 Global transcriptome-wide analysis of CIK cells identify distinct roles of IL-2 and IL-15 in acquisition of cytotoxic capacity against tumor. BMC Medical Genomics, 7,1(2014–08–09) 7: 49. 10.1186/1755-8794-7-49 PMC4134122 25108500

[bib46] Weng C. Dong H. Chen G. Zhai Y. Bai R., 2012 miR-409–3p inhibits HT1080 cell proliferation, vascularization and metastasis by targeting angiogenin. Cancer Lett. 323: 171–179. 10.1016/j.canlet.2012.04.010 22531314

[bib47] Wright G. W. Simon R. M., 2003 A random variance model for detection of differential gene expression in small microarray experiments. Bioinformatics 19: 2448–2455. 10.1093/bioinformatics/btg345 14668230

[bib48] Wu D. Niu X. Tao J. Li P. Lu Q., 2017a MicroRNA-379–5p plays a tumor-suppressive role in human bladder cancer growth and metastasis by directly targeting MDM2. Oncol. Rep. 37: 3502–3508. 10.3892/or.2017.5607 28498468

[bib49] Wu J. Zhu H. Song W. Li M. Liu C., 2014 Identification of conservative microRNAs in Saanen dairy goat testis through deep sequencing. Reprod. Domest. Anim. 49: 32–40. 10.1111/rda.12217 23981187

[bib50] Wu N. Gaur U. Zhu Q. Chen B. Xu Z., 2017b Expressed microRNA associated with high rate of egg production in chicken ovarian follicles. Anim. Genet. 48: 205–216. 10.1111/age.12516 27781291

[bib51] Wylie A. D. Fleming J. A. Whitener A. E. Lekven A. C., 2014 Post-transcriptional regulation of wnt8a is essential to zebrafish axis development. Dev. Biol. 386: 53–63. 10.1016/j.ydbio.2013.12.003 24333179

[bib52] Xie X. Li Y. S. Xiao W. F. Deng Z. H. He H. B., 2017 MicroRNA-379 inhibits the proliferation, migration and invasion of human osteosarcoma cells by targetting EIF4G2. Biosci. Rep. 37: BSR20160542 10.1042/BSR20160542 28381518 PMC5434889

[bib53] Yan N. Lu Y. Sun H. Tao D. Zhang S., 2007 A microarray for microRNA profiling in mouse testis tissues. Reproduction 134: 73–79. 10.1530/REP-07-0056 17641090

[bib54] Zhang J. Qiang L. Wei Z. Li J. Zheng L., 2010 Comparative profiling of genes and miRNAs expressed in the newborn, young adult, and aged human epididymides. Acta Biochim. Biophys. Sin. (Shanghai) 42: 145–153. 10.1093/abbs/gmp116 20119626

[bib55] Zhang L. Ren F. Zhang Q. Chen Y. Wang B., 2008 The TEAD/TEF Family of Transcription Factor Scalloped Mediates Hippo Signaling in Organ Size Control. Dev. Cell 14: 377–387. 10.1016/j.devcel.2008.01.006 18258485 PMC2292673

[bib56] Zhu Z. Li C. Yang S. Tian R. Wang J., 2016 Dynamics of the Transcriptome during Human Spermatogenesis: Predicting the Potential Key Genes Regulating Male Gametes Generation. Sci. Rep. 6: 19069 10.1038/srep19069 26753906 PMC4750114

